# Characterization of DNA Methylation and Screening of Epigenetic Markers in Polycystic Ovary Syndrome

**DOI:** 10.3389/fcell.2021.664843

**Published:** 2021-05-25

**Authors:** Pengbo Cao, Wanting Yang, Peijun Wang, Xihe Li, Buhe Nashun

**Affiliations:** ^1^State Key Laboratory of Reproductive Regulation and Breeding of Grassland Livestock, School of Life Sciences, Inner Mongolia University, Hohhot, China; ^2^Research Center for Animal Genetic Resources of Mongolia Plateau, School of Life Sciences, Inner Mongolia University, Hohhot, China; ^3^Inner Mongolia Saikexing Institute of Breeding and Reproductive Biotechnology in Domestic Animals, Hohhot, China

**Keywords:** PCOS, DNA methylation, miRNA, transcriptome, granulosa cells

## Abstract

Polycystic ovary syndrome (PCOS) is a heterogeneous endocrine and metabolic disorder in women, which is characterized by androgen excess, ovulation dysfunction, and polycystic ovary. Although the etiology of PCOS is largely unknown, many studies suggest that aberrant DNA methylation is an important contributing factor for its pathological changes. In this study, we investigated DNA methylation characteristics and their impact on gene expression in granulosa cells obtained from PCOS patients. Transcriptome analysis found that differentially expressed genes were mainly enriched in pathways of insulin resistance, fat cell differentiation, and steroid metabolism in PCOS. Overall DNA methylation level in granulosa cells was reduced in PCOS, and the first introns were found to be the major genomic regions that were hypomethylated in PCOS. Integrated analysis of transcriptome, DNA methylation, and miRNAs in ovarian granulosa cells revealed a DNA methylation and miRNA coregulated network and identified key candidate genes for pathogenesis of PCOS, including BMP4, ETS1, and IRS1. Our study shed more light on epigenetic mechanism of PCOS and provided valuable reference for its diagnosis and treatment.

## Introduction

Polycystic ovary syndrome (PCOS) is a common endocrine and metabolic disorder. According to the Rotterdam or Androgen Excess and Polycystic Ovary Syndrome diagnosis criteria, PCOS is characterized primarily by hyperandrogenism and accompanied by hirsutism, androgenetic alopecia, and acne (Fauser et al., 2004; Azziz et al., 2009). Other features include polycystic ovarian morphology (PCOM) and ovulation dysfunction, such as oligo-ovulation and anovulation (Qi et al., 2019). PCOS affects approximately 5–20% of women of reproductive age (Fauser et al., 2004). Girls with hereditary PCOS begin to develop hyperinsulinemia as early as age 4 years, and premature pubarche and menstrual irregularity during adolescence affect their health. Postmenopausal women with PCOS have increased risk of cardiovascular and cerebrovascular diseases (Macut et al., 2017). Furthermore, long-term morbidity of PCOS is associated with various complications. Many patients suffer from metabolic syndrome, which increases the prevalence of type 2 diabetes mellitus (T2DM) (Moran et al., 2010; Gambineri et al., 2012) and gestational diabetes (Pan et al., 2015). Some studies also indicated that women with PCOS are more likely to suffer from depression and anxiety (Dokras et al., 2011). Moreover, recent studies have shown that the gut microbiota is changed in individuals with PCOS (Qi et al., 2019).

Genetic and lifestyle factors contribute to pathogenesis of PCOS (Edgar Ricardo et al., 2019). Maternal hyperandrogenism and insulin resistance can be transmitted to offspring, and obesity induced by imbalance of food intake and energy expenditure can lead to increased androgen and influence the severity of insulin resistance, which in turn contribute to the development of PCOS (Carmina, 2003; Merkin et al., 2016). However, no single gene has been identified as the common etiology of PCOS, and recent studies have turned attention to the epigenetic mechanism of PCOS. Multiple genome-wide studies suggested that epigenetic factors, such as non-coding RNAs and DNA methylation, are closely associated with PCOS (Yu Y.Y. et al., 2015; Mu et al., 2021). microRNA is involved in proliferation, apoptosis, and steroid production of ovarian cells (Sirotkin et al., 2010). The miR-513a-3p is expressed in human ovarian granulosa cells (GCs) and plays a central role in ovarian follicular maturation, ovulation, and maintenance of corpus luteum (Liu S. et al., 2015). However, in PCOS patients, miR-99a and miR-323 target insulin-like growth factor 1 receptor (IGF-1R) and IGF-1, respectively, to regulate GC apoptosis (Geng et al., 2019; Wang et al., 2019). In addition, hundreds of lncRNAs were aberrantly expressed in cumulus cells from PCOS patients (Huang et al., 2016), and CTBP1-AS was identified as an androgen-responsive lncRNA that promotes transcriptional activity of androgen receptor and cell cycle progression (Liu et al., 2017).

DNA methylation, as an important content of epigenetics, plays an important role in pathogenesis of PCOS. Studies showed that DNA methylation is increased in the promoter region of the peroxisome proliferator-activated receptor gamma 1 (PPARGC1A) and represses its expression. Reduced PPARGC1A expression is associated with insulin resistance, high serum androgen levels, and reduction of mitochondrial DNA content in women with PCOS (Zhao et al., 2017). Conversely, DNA methylation level of LHCGR gene promoter is reduced in PCOS, and its overexpression leads to increased LH in GCs, which in turn leads to gonadotropin disorder in PCOS women (Mutharasan et al., 2013). In addition, many studies reported that expression of genes involved in cellular processes, such as lipid and steroid synthesis and sugar metabolism, is altered by abnormal DNA methylation and also contributes to the pathogenesis of PCOS (Salehi Jahromi et al., 2018) (Huang et al., 2007; Kokosar et al., 2016).

In this study, we tried to elucidate the epigenetic regulatory mechanism of PCOS by integrating DNA methylation, transcriptome, and miRNA profile in GCs of PCOS. Our study depicted the DNA methylation characteristic of PCOS. Importantly, we predicted a DNA hypomethylation and miRNA coregulated network in PCOS and identified several marker genes using bioinformatics method. The findings of study provide valuable reference for diagnosis and treatment of PCOS.

## Materials and Methods

### Dataset Collection

The data used in this study was downloaded from Gene Expression Omnibus (GEO) (Mao et al., 2021). The RNA-seq data were obtained under accession number GEO: GSE155489. The DNA methylation MBD-seq data were obtained under accession number GEO: GSE138573. The miRNA data sequenced by small RNA-seq were obtained under accession number GEO: GSE138572. The data were generated using GCs obtained from PCOS and normal ovaries. Four duplicate samples of PCOS and control GCs were included in RNA-seq data, respectively. Three duplicate samples of PCOS and control GCs were included in MDB-seq, respectively. Five duplicate samples of PCOS and control GCs were included in miRNA data, respectively.

### Differentially Expressed Genes and Differentially Expressed miRNAs Analysis

Genes with averaged FPKM < 0.1 in all individual samples were removed, and the remaining genes were considered as expressed genes. Similarity, only miRNAs with averaged FPKM > 0.1 in all individual samples were used in the analysis (Cao et al., 2014). R package DEseq2 was used in differentially expressed gene (DEG) analysis and differentially expressed miRNA (DEmiR) analysis, and read count was used as input. Genes and miRNAs with absolute log2 fold change (FC) > 1 and *q* < 0.05 were defined as DEGs and DEmiRs, respectively, where *q* value is the result of *p* value correction (Cao et al., 2020; Ping et al., 2020).

### Analysis of Differentially Methylated Regions

SMART2 package of Python was used for the differentially methylated region (DMR) analysis. The parameter setting was as follows: CpG Distance: 500, AbsMeanMethDiffer: 0.3, p_DMR: 0.05, Euclidean_Distance: 0.2, Segment_Length: 20, and Methylation_Specificity: 0.5 (Liu H. et al., 2015). DNA methylation level of the gene was represented by average DNA methylation level of CpG segments in gene promoters, and | Δβ| > 0.2 was used to define differentially methylated gene (Gao et al., 2018). Hypermethylation and hypomethylation marks of each group were determined by the one-sample *t* test in SMART2.

### PPI Network Analysis

DNA hypomethylation-affected upregulated genes were identified by integrated analysis of transcriptome and DNA methylation. The human protein interaction pairs (9606.protein.links.v11.0) were downloaded from the STRING database^1^ and used as background network. The pairwise interaction genes in the DNA hypomethylation regulated gene modules were defined as seed nodes, and the Pearson correlation coefficient was used as the weight of the edges. By combining the background PPI network and the co-expression networks composed of the seed nodes, two gene co-expression networks of candidate markers were obtained (Mao et al., 2021). MCODE application was used for models mining. The parameters settings of MCODE application were as follows: degree cutoff: 2, node score cutoff: 0.2, K-Core: 2, and maximum depth: 100.

### Construction of miRNAs Target Gene Network

The target genes of DEmiRs were identified using TarBase database (7.0)^2^ (Vlachos et al., 2015). The intersection of miRNA target genes and DEGs between PCOS and controls were considered as genes that were regulated by miRNA in PCOS.

### Gene Ontology and Kyoto Encyclopedia of Genes and Genomes Pathway Analysis

Functional annotation was performed with the DAVID database^3^ (Dennis et al., 2003; Sun et al., 2020). Gene Ontology (GO) terms and Kyoto Encyclopedia of Genes and Genomes (KEGG) pathways for each functional cluster were summarized to a representative term, and *p* values were plotted to show the significance (Bao et al., 2019, 2021).

### Statistical Analysis and Data Visualization

The R/Bioconductor software packages^4^ and Python package were used in the statistical analysis (ChiPseeker, dplyr, psych add SMART2) (Yu G. et al., 2015; Reece and Hulse, 2020). All networks were visualized using the software Cytoscape (Shannon et al., 2003). R packages (pheatmap, UpSetR, CMplot, ggplot2, and GOplot) were used for data visualization [heatmap, upset plot, Manhattan plot, principal component analysis (PCA) plot, Go chord plot] (Conway et al., 2017; Ni et al., 2019; Cao et al., 2020; Zhou et al., 2020).

## Results

### Transcriptional Profiles of GCs in Human PCOS

PCOS is a metabolic disease whose etiology has not been fully understood (Li S. et al., 2016). Accumulating evidences suggest that abnormal gene expression is one of the main contributing factors for the development of PCOS. In order to take a deeper insight into the potential molecular mechanism of PCOS, we conducted genome-wide transcriptome analysis based on RNA-seq data in GCs obtained from PCOS and normal ovaries.

**FIGURE 1:**
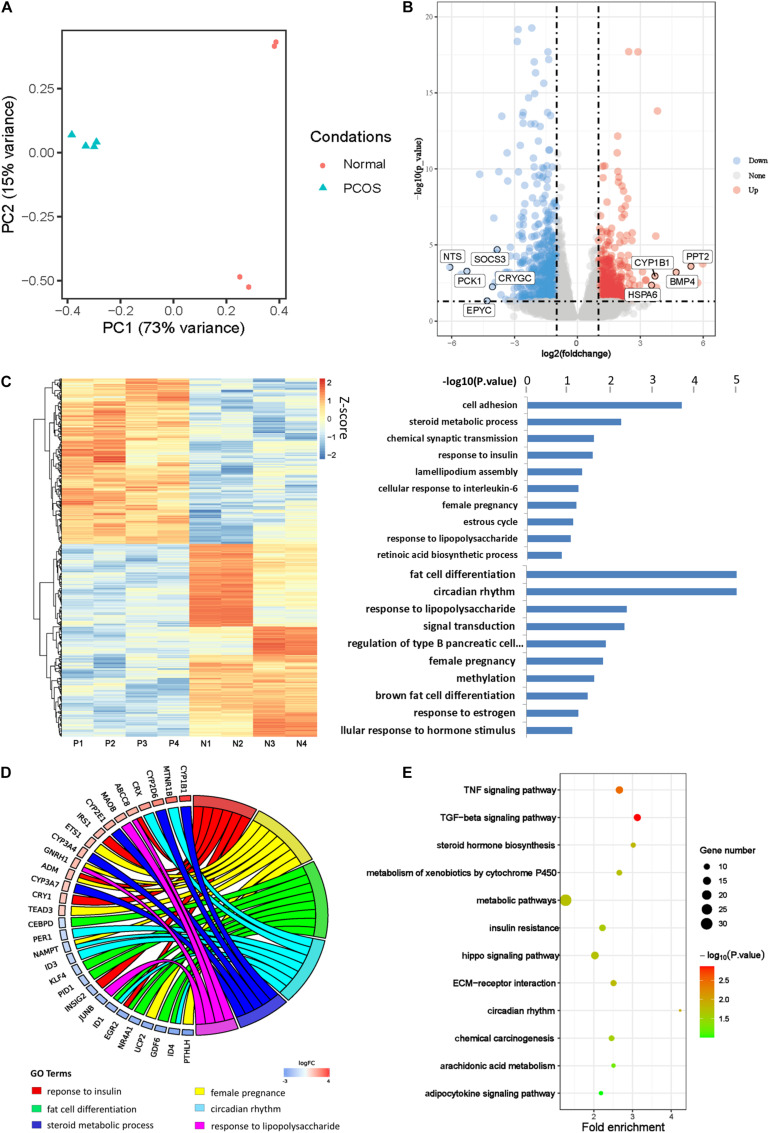
Transcriptional profile of human PCOS. **(A)** Principal component analysis (PCA) of global transcriptomes in GCs obtained from PCOS and control ovaries. The samples are represented by different colors as indicated in the right. **(B)** Volcano plot showing differentially expressed genes (DEGs) obtained by a pairwise comparison between PCOS and control GCs. Upregulated and downregulated genes were colored in red and blue, respectively. **(C)** Analysis of PCOS transcript profiles and genes function. **(D)** The GO chord showing the biological processes and genes that participate in PCOS. **(E)** KEGG analysis of differentially expressed genes between PCOS and control.

PCA was first performed to investigate the transcriptional difference between GCs of PCOS and normal ovaries (Figure 1A). The PC1 axis contributed to the main difference between PCOS and normal GCs, accounting for 73%. On this axis, PCOS and normal GCs were split into two separate clusters showing distinct gene expression profiles. The control samples did not gather together on PC2 axis, which may due to the heterogeneity of normal individuals. This finding was consistent with the clustering of sample correlation coefficient analysis (Supplementary Figure 1A). We further identified 470 upregulated genes and 548 downregulated genes in GCs from PCOS (*p*_*adj*_ < 0.05 and |log2FC| > 1; Figure 1B). Among them, PCK1 is the rate-limiting enzyme that regulates gluconeogenesis, and CYP1B1 plays an important role in estrogen metabolism, probably corresponding to the phenotype of obesity and hormonal disturbance in PCOS patients, respectively.

Then we performed unsupervised hierarchical clustering analysis of these DEGs and confirmed a significantly different gene expression pattern between PCOS and the controls (Figure 1C). We further conducted GO analysis and found the DEGs were significantly enriched in biological processes such as steroid metabolic process, response to insulin, female pregnancy, estrous cycle, response to lipopolysaccharide, and fat cell differentiation (Figure 1C), implying that abnormal transcriptional changes in these biological processes may contribute to the development of PCOS. In support of our hypothesis, some genes involved in these biological processes have already been reported to play important roles in PCOS pathogenesis. For example, nuclear receptor subfamily 4 group A member 1 (NR4A1) is upregulated in ovarian GCs and participates in upregulation of androgen in PCOS patients (Song et al., 2019). CEBPD is a leucine zipper transcription factor involved in inflammation and adipogenesis in PCOS (Ma et al., 2020; Figure 1D). Except for the reported findings, we found one set of genes including ID1, ID2, and ID3 was significantly enriched in biological processes of the circadian rhythm and speculated that insomnia in some PCOS patients may due to the abnormal expression of these genes. In order to take a closer look at the changes in signaling pathways, we carried out KEGG analysis and found that the DEGs were enriched in signaling pathways of tumor necrosis factor (TNF), transforming growth factor β (TGF-β), and metabolic and steroid hormone biosynthesis (Figure 1E). These results suggested that the biological processes and signaling pathways in which the DEGs are enriched could contribute to the development of PCOS.

### The Methylome Profile of GCs in Human PCOS

**FIGURE 2:**
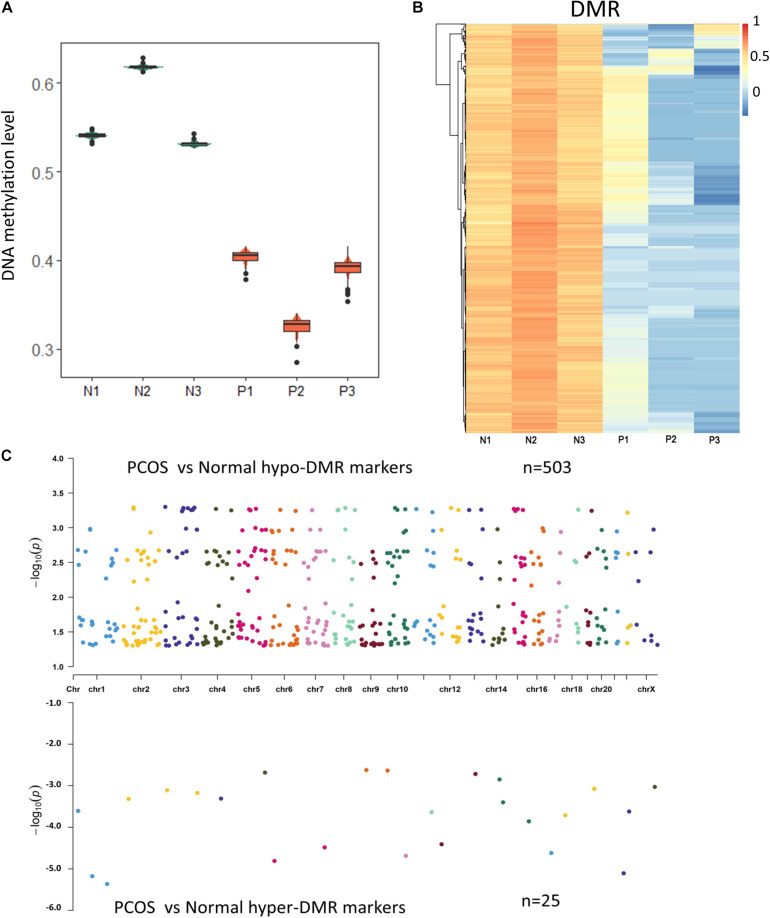
The methylome changes of human PCOS. **(A)** Violin plot of DNA methylation levels of PCOS and control GCs. **(B)** Heatmap of DMRs between PCOS and control GCs. Each row represents a DMR, and averaged CpG methylation levels are represented by different colors. **(C)** Chromosome distribution of DMRs. The number of dots represents the distribution of DMR across different chromosomes.

**FIGURE 3:**
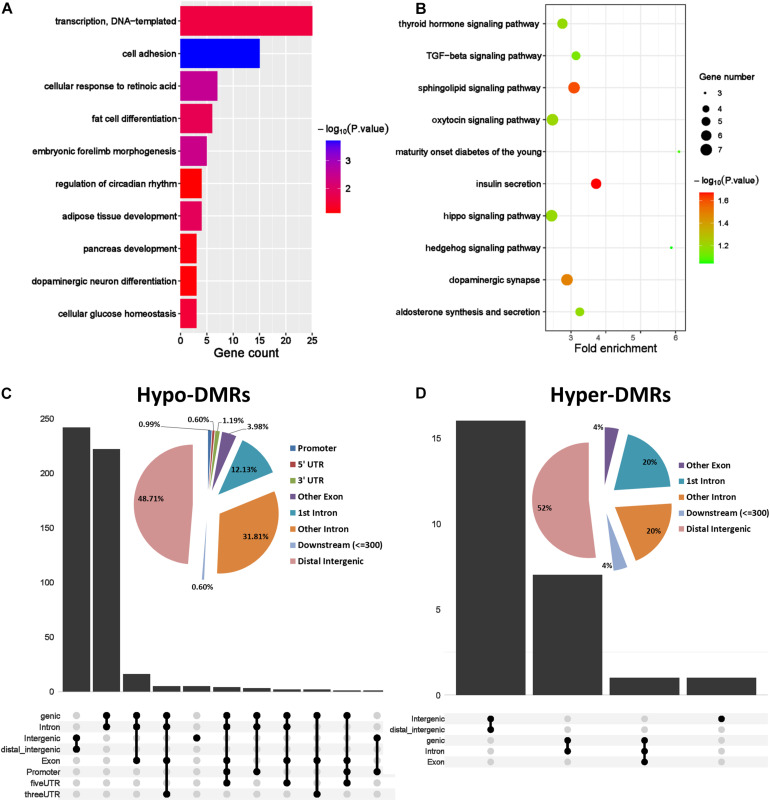
Genomic region preference and functional analysis of DMRs. **(A)** GO term analysis of hypo DMRs in GCs obtained from PCOS and control ovaries. **(B)** KEGG analysis of hypo DMRs in GCs obtained from PCOS and control ovaries. **(C,D)** Genomic distribution of the hypo DMRs and hyper DMRs. Pie charts represent proportion of DMRs in different genomic contexts. Upset graphs represent the number of DMRs distributed in single or combined genomic regions.

Previous studies have shown that epigenetic changes including DNA methylation are crucial for the development of PCOS. To characterize the abnormal DNA methylation in PCOS, we evaluated overall DNA methylation levels and found remarkable hypomethylation in GCs from PCOS compared with the normal GCs (Figure 2A). In order to take a closer look at the potential regulatory mechanism of aberrant DNA methylation in PCOS, we performed DMR analysis using Python package “SMART2” (Figure 2B) and identified hyper-DMRs and hypo-DMRs, respectively (Figure 2C). The hypo-DMRs accounted for the majority of DMRs (*n* = 495), whereas there were only 25 hyper-DMRs that account for 5% of the DMRs. These results indicated that hypomethylation is a key characteristic of PCOS and prompted us to focus on it. GO analyses found that the hypo-DMRs genes were mainly enriched in GO terms of adipose tissue development, glucose homeostasis, and pancreas development, which correspond to the characteristics of acne, obesity, and insulin resistance in PCOS patients (Figures 3A,B). In the KEGG enrichment analysis, the hypo-DMRs genes were found to be mainly involved in insulin secretion, diabetes, dopaminergic synapse, and thyroid hormone signaling pathways, which may associate with the diabetes and hormonal disorders in PCOS patients. Taken together, hypo-DMR plays a critical role in PCOS by altering gene expression in important biological processes and signaling pathways.

In order to investigate the genomic regional preference, the DMRs were mapped to the whole genome. Distribution map of DMRs showed that hypo-DMRs were more prevalent in promoter region near TSS than hyper-DMRs (Supplementary Figures 1C,D). Interestingly, besides the longer genomic regions such as the distal intergenic regions and other intron regions, the DMRs were predominantly located in the first intron. This suggested that DNA hypomethylation in the first intron probably plays an important role in regulating gene expression in PCOS (Figures 3C,D).

### Integrated Analysis of DNA Methylation and Transcriptome in PCOS

**FIGURE 4:**
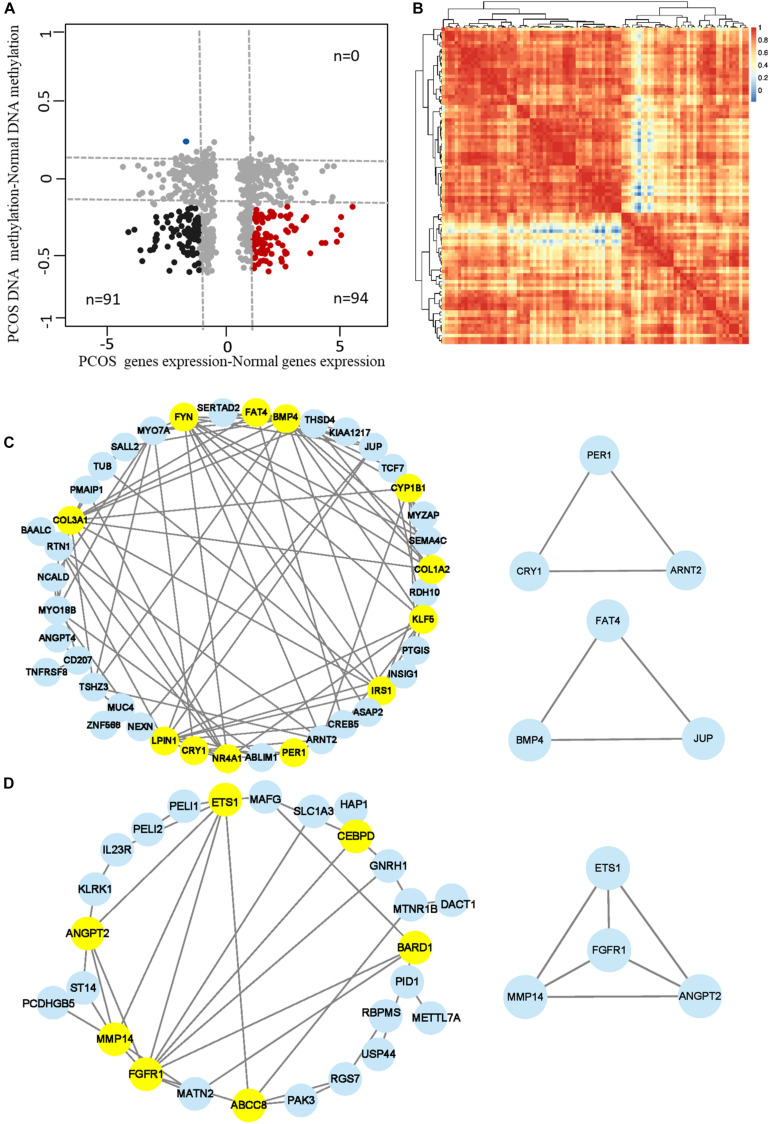
Identification of the hub methylation marker genes in PCOS. **(A)** Scatterplots showing the comparison between transcriptome and the DNA methylome. Genes with significant changes in DNA methylation levels (abs change >0.2) and expression levels (abs log2 fold change >1) were defined as “methylation-affected genes.” Hypermethylation-affected downregulated genes were labeled in blue; Hypomethylation-affected upregulated genes were labeled in red. **(B)** Heatmap showing the module clustering of 94 methylation-affected upregulated genes by Pearson correlation coefficient. **(C,D)** Network diagrams showing interaction of genes in the two modules that were obtained in panel **(B)**. Hub node genes were highlighted by yellow. The genes in the small networks are highly connected in the networks calculated by module mining.

To further explore the role of aberrant DNA methylation in PCOS, we carried out an integrated analysis of the transcriptome and DNA methylome. The genes affected by abnormal DNA methylation were identified by comparing changes in gene expression and methylation levels between PCOS and the normal GCs. Genes with significant changes in DNA methylation levels (abs change >0.2) and expression levels (abs log2 FC > 1) were defined as “methylation-affected genes” (Figure 4A). All genes were divided into methylation-affected genes with either repressed or activated expression. As DNA hypomethylation is an important characteristic of GCs in PCOS and DNA methylation inversely associates with gene expression, we identified a subset of hypomethylation-affected upregulated genes (*n* = 94) and calculated the Pearson correlation coefficient between gene pairs and performed unsupervised hierarchical clustering (Figure 4A). Then, two closely related gene modules with strong correlations were generated for subsequent analysis (Figure 4B). Thus, our analysis identified a number of important PCOS-related genes whose expression level was regulated by DNA hypomethylation.

### Analysis of Gene Coexpression Network

Based on the pairwise interaction genes in the aforementioned two modules (Figure 4B) and human–protein interaction pairs (9606.protein.links.v11.0) downloaded from the STRING database, two-gene coexpression interaction networks were constructed by Cytoscape (Figures 4C,D). Based on topological properties of the networks, genes with high degree of node (hub node genes) were identified as candidate genes. Then, three small modules composed of 10 highly connected genes were extracted from the two networks using the Cytoscape application “MCODE” (Figures 4C,D). The genes in these modules were considered as marker genes regulated by hypomethylation, including BMP4, KLF5, PER1, EST1, CRY1, and FAT4. Some of the candidate genes have been reported to play key roles in PCOS such as ETS1 and NR4A1 (Kasch et al., 2018; Song et al., 2019). Among the other genes, BMP4 regulates conversion of white and brown fat and is closely related to the occurrence of T2DM (Hoffmann et al., 2017); the circadian gene PER1 senses progesterone signal during human endometrial decidualization (Zhang et al., 2019). These genes may be involved in the development of PCOS by regulating insulin metabolism, adipocyte differentiation, and circadian biological processes. In general, we constructed two-gene coexpressed networks of PCOS and identified several PCOS-related target markers regulated by DNA hypomethylation.

### The miRNAs Profiles of GCs in Human PCOS

**FIGURE 5:**
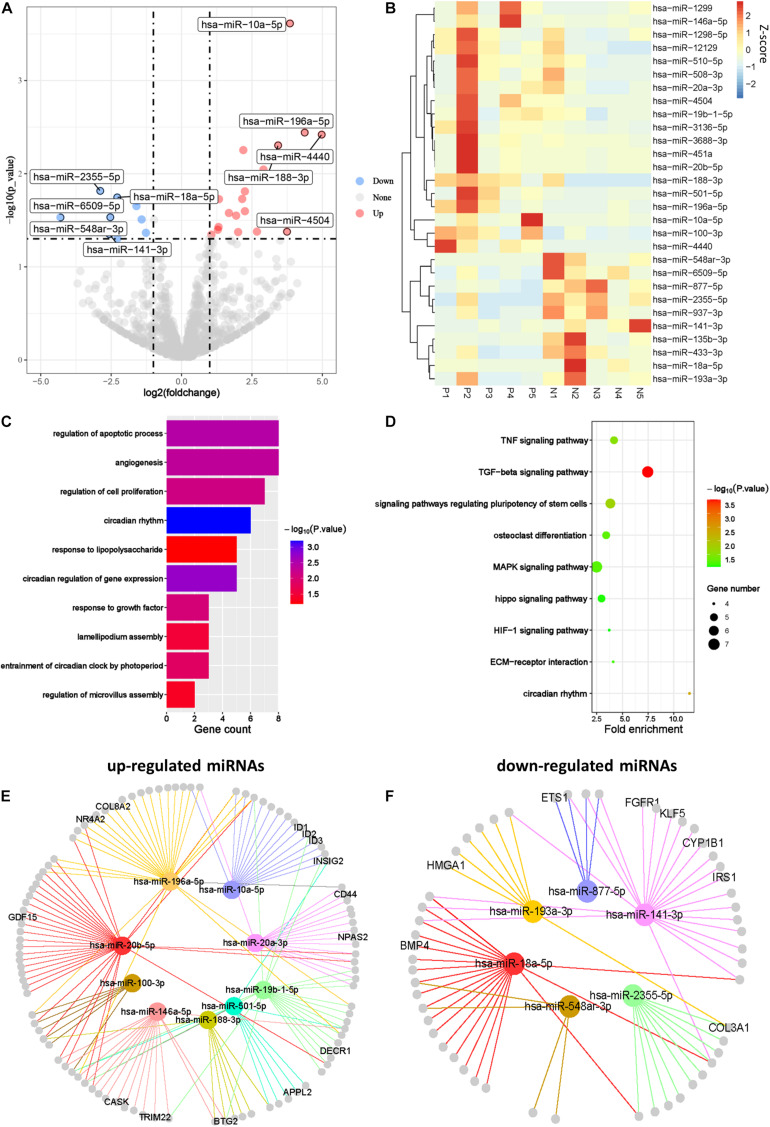
The miRNA profiles of Human PCOS. **(A)** Volcano plot comparing miRNA expression profiles of PCOS and control GCs. Upregulated and downregulated miRNAs were colored in red and blue, respectively. **(B)** Heatmap of differentially expressed miRNAs between PCOS and control GCs. Each row represents a miRNA, and colors represent expression levels. **(C,D)** GO and KEGG analysis of differentially expressed miRNA target genes GCs obtained from PCOS and control ovaries. **(E)** Network diagram showing interaction between upregulated miRNAs and their target genes. **(F)** Network diagram showing interaction between downregulated miRNAs and their target genes. Colored nodes represent miRNA; gray nodes represent target genes; colored edges indicate miRNA-target interaction.

Abnormal expression of miRNAs in female reproductive organs such as uterus, fallopian tubes, and ovaries is involved in pathological changes of the organs (Bagga et al., 2005). To illustrate the regulatory functions of miRNAs in PCOS, we compared miRNA profiles of GCs in PCOS and normal ovaries. We identified 19 upregulated miRNAs and 10 downregulated miRNAs in PCOS, respectively (*p*_*adj*_ < 0.05 and |log2FC| > 1; Figure 5A). Unsupervised hierarchical clustering analysis of the DEmiRs showed distinct expression patterns in the PCOS and control GCs (Figure 5B). Of note, miR-141-3p was one of the key miRNA significantly downregulated in PCOS, which was reported to inhibit cell proliferation and promotes apoptosis (Lin et al., 2014). Consistent with previous report in cumulus cells (Liu S. et al., 2015), miR-508-3p was upregulated in GCs of PCOS patients. In order to further explore the function of miRNAs in PCOS, we performed GO and KEGG enrichment analysis on the target genes of the DEmiRs. The target genes of the DEmiRs were mainly enriched in GO terms of circadian rhythm, apoptotic process, cell proliferation, and lipopolysaccharide. In the KEGG enrichment analysis, the target genes were mainly involved in circadian rhythm, TGF-β signaling pathway, and TNF signaling pathway (Figures 5C,D). These results indicated that the DEmiRs contribute to the development of PCOS by regulating some key biological processes and signaling pathways.

It is well known that miRNA can lead to the degradation or translational inhibition of mRNA (Bartel, 2004). Taking the intersection of miRNA target genes and DEGs between PCOS and controls, we identified genes that were most likely regulated by miRNA in PCOS, including upregulated miRNA target genes (*n* = 106) and downregulated miRNA target genes (*n* = 56), respectively (Supplementary Figure 1E). In order to elucidate possible regulatory function of miRNAs in gene expression, we performed miRNA–gene interaction networks analysis using Cytoscape (Figures 5E,F). Some of the genes in the networks, such as GDF15, INSIG2, have already been reported to be involved in PCOS. For example, GDF15 is closely related to insulin resistance, hyperandrogenemia, and menstrual disorder in PCOS (Berberoglu et al., 2015). Apart from the reported genes, we newly identified one set of genes that were represented by CD44, IRSI, CYP1B1, and HMGA1. These genes play important roles in ovarian function and androgen metabolism and are likely to be involved in pathogenesis of PCOS. For example, the deficiency of endometrial epithelial CD44 adhesion complex contributes to the endometrial infertility (Paravati et al., 2020) and likely to be involved in the pathogenesis of PCOS by affecting ovarian function. In conclusion, we constructed the miRNAs regulatory networks of PCOS and identified several important target genes regulated by aberrant miRNAs.

**FIGURE 6:**
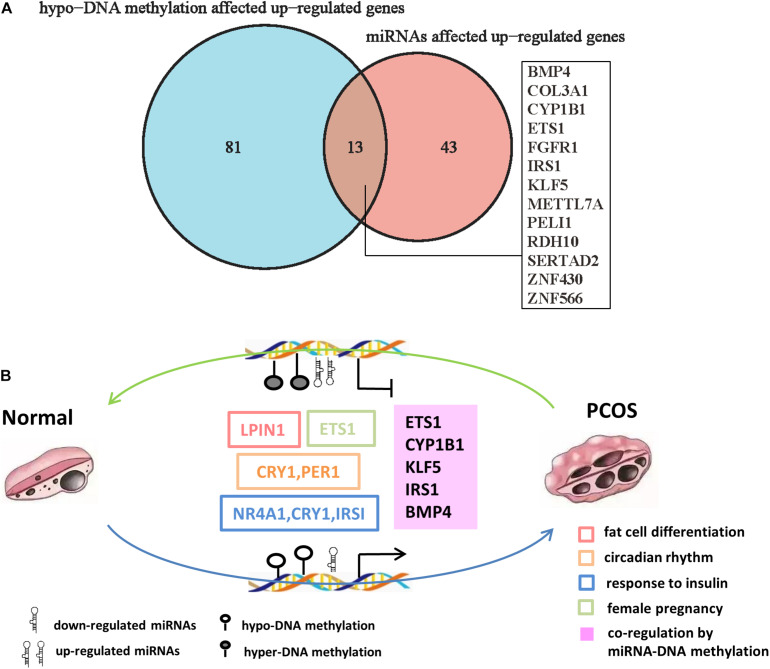
The DNA methylation and miRNA coregulation profile in PCOS. **(A)** Venn diagram showing the genes that are coregulated by downregulated miRNAs and reduced DNA methylation in PCOS. **(B)** Coregulation of DNA methylation and miRNA in PCOS. The key molecular markers and pathways are labeled in different colors. The hollow box represents the signaling pathway where the marker genes are involved, and the solid box represents the genes that are coregulated by miRNA and DNA methylation.

Importantly, we found 13 genes including BMP4, ETS1, IRS1, FGFR1, CYP1B1, and KLF5, which were coregulated by downregulated miRNAs and hypo-DNA methylation (Figure 6A). Some of the genes such as ETS1 and FGFR1 have been reported to participate in the development of PCOS (Song et al., 2019; Patil et al., 2020). Among the other genes, KLF5 is an important transcription factor that regulates androgen-AR signaling (Li et al., 2020); CYP1B1, a dioxin-inducible oxidoreductase, is involved in the metabolism of estradiol (Muneeb et al., 2014); IRS1 is an insulin receptor substrate gene that mediates the control of various cellular processes by insulin (Kasch et al., 2018; Park et al., 2018). These genes were significantly enriched in biological processes of adipocyte differentiation, insulin resistance, female pregnancy, and circadian rhythm, indicating they are likely to be involved in pathogenesis of PCOS. Taken together, our analysis predicted a DNA methylation and miRNA-mediated coregulation profile in PCOS (Figure 6B).

## Discussion

Accumulating evidences indicated that epigenetic alterations occur in the peripheral and umbilical cord blood, as well as in ovary, adipose tissue, and skeletal muscle in women with PCOS (Sirotkin et al., 2010; Roth et al., 2014; Edgar Ricardo et al., 2019). These epigenetic alternations are correlated with systemic and tissue-specific dysfunctions in PCOS, highlighting their importance in PCOS pathogenesis (Edgar Ricardo et al., 2019). As GCs establish a very close relationship with the female gametes even before oogonia differentiation and better represent molecular characteristics of PCOS, we focused on ovarian GCs to investigate the epigenetic regulatory mechanism of PCOS.

Many previous studies suggested that lifestyle and genetic factors contribute to the development of PCOS (Li S. et al., 2016). However, the etiology of PCOS remains unclear. In this study, we investigated DNA methylation characteristics and screened potential epigenetic markers. We identified several key genes, such as IRS1, KLF5, CYP1B1, ETS1, and BMP4, and some signaling pathways including steroid metabolic process, response to insulin, female pregnancy, estrous cycle, and fat cell differentiation. The alternation of these genes’ expression and signaling pathways could contribute to the symptoms of PCOS, such as the type 2 diabetes, infertility, hormonal disorders, and obesity (Combs et al., 2021; Mao et al., 2021). Additionally, we found some DEGs were significantly enriched in circadian rhythm signaling pathway (Figure 1E), suggesting that insomnia observed in some of the PCOS patients might be due to the expression changes of these genes.

Change in DNA methylation is involved in various diseases processes, and aberrant DNA methylation of CYP17, CEBPB, PPARG, and SVEP1 genes has been reported in PCOS (Huang et al., 2007; Kokosar et al., 2016). Consistent with previous studies, we found that overall DNA methylation is reduced in GCs of PCOS (Liu et al., 2020) and further identified several marker genes that are regulated by the hypo-DNA methylation, including BMP4, KLF5, IRS1, LPIN1, and ABCC8. Among them, LPIN1 plays a critical role in adipocyte differentiation and lipid metabolism (Chang et al., 2010). Pathogenic variants of ABCC8 are the most common genetic cause of neonatal diabetes and hyperinsulinism (De Franco et al., 2020). Furthermore, these genes were enriched in biological processes of insulin and lipid metabolism, and dysregulation of these genes may contribute to the development of PCOS. Interestingly, the first intron was found to be the main genomic region where DNA hypomethylation happened. As DNA methylation of the first intron inversely associates with gene expression regardless of tissue and species (Anastasiadi et al., 2018), reduction of DNA methylation in the first intron could lead to upregulation in gene expression and disturb normal biological process. This indicated that the first intron was an important genomic functional region whose change in DNA methylation likely contributes to the pathogenesis of PCOS.

Abnormal activation or inhibition of signal pathway is closely related to the development of PCOS. TGF-β signaling pathway and mitogen-activated protein kinase (MAPK) signaling pathway have been reported to be involved in PCOS (Liu S. et al., 2015). In our study, we found the DEmiRs were enriched in TGF-β signaling pathway, MAPK signaling pathway, and circadian rhythm. These findings collectively suggested that TGF-β and MAPK signaling pathway are crucial for the development of PCOS and supported our proposal that miRNAs regulation is an important layer of regulatory machinery in PCOS. miR-141-3p was reported as an important DEmiR in the rat model of PCOS (Li D. et al., 2016) and found to regulate target genes such as KLF5, IRS1, and CYP1B1 in GCs of PCOS patients in our study (Figure 5F). Coincidentally, DNA hypomethylation happens on these target genes, and their expression was upregulated (Figure 6A), indicating a coregulatory mechanism of DNA hypomethylation and miRNA in the pathogenesis of PCOS.

In conclusion, our study indicated that DNA hypomethylation is one of the main characteristics of PCOS, and the first intron was found to be the key genomic elements where DNA hypomethylation was observed, indicating its active involvement in the pathogenesis of PCOS. Importantly, we predicted a DNA hypomethylation and miRNA coregulated network in PCOS and provided several candidate target genes including BMP4, CYP1B1, IRS1, ETS1, and LPIN1. These genes participate in important signaling pathways and biological process and potentially serve as molecular targets for diagnosis and treatment of PCOS. However, our findings are based on genome-wide data analysis and need further validation in experimental models. As developmental competence of oocyte is greatly impaired in PCOS (Alexandria and Jennifer, 2019), it is interesting to investigate the underlying molecular mechanism of oocyte dysfunction in PCOS and clarify its impact on offspring in the future.

## Data Availability Statement

The datasets presented in this study can be found in online repositories. The names of the repository/repositories and accession number(s) can be found in the article/Supplementary Material.

## Author Contributions

BN and PC conceived the study. PC performed the bioinformatics analysis. BN wrote the manuscript with assistance from PC, WY, PW, and XL. All authors contributed to the article and approved the submitted version.

## Conflict of Interest

The authors declare that the research was conducted in the absence of any commercial or financial relationships that could be construed as a potential conflict of interest.

## Footnotes

1 https://string-db.org/

2 http://diana.cslab.ece.ntua.gr/tarbase/

3 https://david.ncifcrf.gov/home.jsp

4 http://www.bioconductor.org

## References

[B1] Alexandria P. S. Jennifer R. W. (2019). Obesity induces ovarian inflammation and reduces oocyte quality. *Reproduction* 158 R79–R90. 10.1530/REP-18-0583 30999278

[B2] Anastasiadi D. Esteve-Codina A. Piferrer F. (2018). Consistent inverse correlation between DNA methylation of the first intron and gene expression across tissues and species. *Epigenetics Chromatin* 11:37. 10.1186/s13072-018-0205-1 29958539 PMC6025724

[B3] Azziz R. Carmina E. Dewailly D. Diamanti-Kandarakis E. Escobar-Morreale H. F. Futterweit W. (2009). The Androgen Excess and PCOS Society criteria for the polycystic ovary syndrome: the complete task force report. *Fertil. Steril.* 91 456–488. 10.1016/j.fertnstert.2008.06.035 18950759

[B4] Bagga S. Bracht J. Hunter S. Massirer K. Holtz J. Eachus R. (2005). Regulation by let-7 and lin-4 miRNAs Results in Target mRNA Degradation. *Cell* 122 553–563. 10.1016/j.cell.2005.07.031 16122423

[B5] Bao S. Hu T. Liu J. Su J. Zhou M. (2021). Genomic instability-derived plasma extracellular vesicle-microRNA signature as a minimally invasive predictor of risk and unfavorable prognosis in breast cancer. *J. Nanobiotechnol.* 19:22. 10.1186/s12951-020-00767-3 33436002 PMC7802300

[B6] Bao S. Zhao H. Yuan J. Fan D. Zhou M. (2019). Computational identification of mutator-derived lncRNA signatures of genome instability for improving the clinical outcome of cancers: a case study in breast cancer. *Brief. Bioinform.* 21 1742–1755. 10.1093/bib/bbz118 31665214

[B7] Bartel D. P. (2004). MicroRNAs: genomics, Biogenesis, Mechanism, and Function. *Cell* 116 281–297. 10.1016/S0092-8674(04)00045-5 14744438

[B8] Berberoglu Z. Aktas A. Fidan Y. Yazici A. C. Aral Y. (2015). Association of plasma GDF-9 or GDF-15 levels with bone parameters in polycystic ovary syndrome. *J. Bone Miner. Metab.* 33 101–108. 10.1007/s00774-013-0560-8 24430093

[B9] Cao P. Li H. Zuo Y. Nashun B. (2020). Characterization of DNA Methylation Patterns and Mining of Epigenetic Markers During Genomic Reprogramming in SCNT Embryos. *Front. Cell Dev. Biol.* 8:570107. 10.3389/fcell.2020.570107 32984351 PMC7492385

[B10] Cao S. Han J. Wu J. Li Q. Liu S. Zhang W. (2014). Specific gene-regulation networks during the pre-implantation development of the pig embryo as revealed by deep sequencing. *BMC Genomics* 15:4. 10.1186/1471-2164-15-4 24383959 PMC3925986

[B11] Carmina E. (2003). Genetic and environmental aspect of polycystic ovary syndrome. *J. Endocrinol. Invest.* 26 1151–1159. 10.1007/BF03345266 15008257

[B12] Chang Y.-C. Chang L.-Y. Chang T.-J. Jiang Y.-D. Lee K.-C. Kuo S.-S. (2010). The Associations of LPIN1 Gene Expression in Adipose Tissue With Metabolic Phenotypes in the Chinese Population. *Obesity* 18 7–12. 10.1038/oby.2009.198 19543209

[B13] Combs J. C. Hill M. J. Decherney A. H. (2021). Polycystic Ovarian Syndrome Genetics and Epigenetics. *Clin. Obstet. Gynecol.* 64 20–25. 10.1097/GRF.0000000000000581 33306497 PMC7855879

[B14] Conway J. R. Lex A. Gehlenborg N. (2017). UpSetR: an R package for the visualization of intersecting sets and their properties. *Bioinformatics* 33 2938–2940. 10.1093/bioinformatics/btx364 28645171 PMC5870712

[B15] De Franco E. Saint-Martin C. Brusgaard K. Knight Johnson A. E. Aguilar-Bryan L. Bowman P. (2020). Update of variants identified in the pancreatic β-cell KATP channel genes KCNJ11 and ABCC8 in individuals with congenital hyperinsulinism and diabetes. *Hum. Mutat.* 41 884–905. 10.1002/humu.23995 32027066 PMC7187370

[B16] DennisG.ShermanB.HosackD.YangJ.LempickiR. (2003). DAVID: database for Annotation, Visualization, and Integrated Discovery. *Genome Biol.* 4:P3. 10.1186/gb-2003-4-5-p312734009

[B17] Dokras A. Clifton S. Futterweit W. Wild R. (2011). Increased risk for abnormal depression scores in women with polycystic ovary syndrome: a systematic review and meta-analysis. *Obstet. Gynecol.* 117 145–152. 10.1097/AOG.0b013e318202b0a4 21173657

[B18] Edgar Ricardo V.-M. Yadira Inés G.-V. Elizabeth G.-G. Christian R.-M. Enrique R.-M. Ignacio C.-A. (2019). DNA methylation in the pathogenesis of polycystic ovary syndrome. *Reproduction* 158 R27–R40. 10.1530/REP-18-0449 30959484

[B19] Fauser B. C. J. M. Chang J. Azziz R. Legro R. Dewailly D. Franks S. (2004). Revised 2003 consensus on diagnostic criteria and long-term health risks related to polycystic ovary syndrome (PCOS). *Hum. Reprod.* 19 41–47. 10.1093/humrep/deh098 14688154

[B20] Gambineri A. Patton L. Altieri P. Pagotto U. Pizzi C. Manzoli L. (2012). Polycystic ovary syndrome is a risk factor for type 2 diabetes: results from a long-term prospective study. *Diabetes* 61 2369–2374. 10.2337/db11-1360 22698921 PMC3425413

[B21] Gao R. Wang C. Gao Y. Xiu W. Chen J. Kou X. (2018). Inhibition of Aberrant DNA Re-methylation Improves Post-implantation Development of Somatic Cell Nuclear Transfer Embryos. *Cell Stem Cell* 23 426–435.e5. 10.1016/j.stem.2018.07.017 30146410

[B22] Geng Y. Sui C. Xun Y. Lai Q. Jin L. (2019). MiRNA-99a can regulate proliferation and apoptosis of human granulosa cells via targeting IGF-1R in polycystic ovary syndrome. *J. Assist. Reprod. Genet.* 36 211–221. 10.1007/s10815-018-1335-x 30374732 PMC6420594

[B23] Hoffmann J. M. Grünberg J. R. Church C. Elias I. Palsdottir V. Jansson J.-O. (2017). BMP4 Gene Therapy in Mature Mice Reduces BAT Activation but Protects from Obesity by Browning Subcutaneous Adipose Tissue. *Cell Rep.* 20 1038–1049. 10.1016/j.celrep.2017.07.020 28768190

[B24] Huang A. M. Rudelius M. Sharan S. McAllister J. M. Raffeld M. Christenson L. K. (2007). The Cebpd (C/EBPdelta) gene is induced by luteinizing hormones in ovarian theca and interstitial cells but is not essential for mouse ovary function. *PLoS One* 2:e1334. 10.1371/journal.pone.0001334 18092000 PMC2129115

[B25] Huang X. Hao C. Bao H. Wang M. Dai H. (2016). Aberrant expression of long noncoding RNAs in cumulus cells isolated from PCOS patients. *J. Assist. Reprod. Genet.* 33 111–121. 10.1007/s10815-015-0630-z 26650608 PMC4717141

[B26] Kasch J. Kanzleiter I. Saussenthaler S. Schürmann A. Keijer J. van Schothorst E. (2018). Insulin sensitivity linked skeletal muscle Nr4a1 DNA methylation is programmed by the maternal diet and modulated by voluntary exercise in mice. *J. Nutr. Biochem.* 57 86–92. 10.1016/j.jnutbio.2018.03.015 29680662

[B27] Kokosar M. Benrick A. Perfilyev A. Fornes R. Nilsson E. Maliqueo M. (2016). Epigenetic and Transcriptional Alterations in Human Adipose Tissue of Polycystic Ovary Syndrome. *Sci. Rep.* 6:22883. 10.1038/srep22883 26975253 PMC4791632

[B28] Li D. Li C. Xu Y. Xu D. Li H. Gao L. (2016). Differential Expression of microRNAs in the Ovaries from Letrozole-Induced Rat Model of Polycystic Ovary Syndrome. *DNA Cell Biol.* 35 177–83. 10.1089/dna.2015.3145 26745201

[B29] Li J. Zhang B. Liu M. Fu X. Ci X. A J. (2020). KLF5 Is Crucial for Androgen-AR Signaling to Transactivate Genes and Promote Cell Proliferation in Prostate Cancer Cells. *Cancers* 12:748. 10.3390/cancers12030748 32245249 PMC7140031

[B30] Li S. Zhu D. Duan H. Tan Q. (2016). The epigenomics of polycystic ovarian syndrome: from pathogenesis to clinical manifestations. *Gynecol. Endocrinol.* 32 942–946. 10.1080/09513590.2016.1203409 27425146

[B31] Lin L. Liang H. Wang Y. Yin X. Hu Y. Huang J. (2014). microRNA-141 inhibits cell proliferation and invasion and promotes apoptosis by targeting hepatocyte nuclear factor-3β in hepatocellular carcinoma cells. *BMC Cancer* 14:879. 10.1186/1471-2407-14-879 25425543 PMC4289273

[B32] Liu H. Liu X. Zhang S. Lv J. Li S. Shang S. (2015). Systematic identification and annotation of human methylation marks based on bisulfite sequencing methylomes reveals distinct roles of cell type-specific hypomethylation in the regulation of cell identity genes. *Nucleic Acids Res.* 44 75–94. 10.1093/nar/gkv1332 26635396 PMC4705665

[B33] Liu S. Zhang X. Shi C. Lin J. Chen G. Wu B. (2015). Altered microRNAs expression profiling in cumulus cells from patients with polycystic ovary syndrome. *J. Transl. Med.* 13:238. 10.1186/s12967-015-0605-y 26198660 PMC4508762

[B34] Liu L. He D. Wang Y. Sheng M. (2020). Integrated analysis of DNA methylation and transcriptome profiling of polycystic ovary syndrome. *Mol. Med. Rep.* 21 2138–2150. 10.3892/mmr.2020.11005 32323770 PMC7115196

[B35] Liu Y.-d Li Y. Feng S.-X. Ye D.-S. Chen X. Zhou X.-Y. (2017). Long Noncoding RNAs: potential Regulators Involved in the Pathogenesis of Polycystic Ovary Syndrome. *Endocrinology* 158 3890–3899. 10.1210/en.2017-00605 28938484

[B36] Ma S. Sun S. Geng L. Song M. Wang W. Ye Y. (2020). Caloric Restriction Reprograms the Single-Cell Transcriptional Landscape of Rattus Norvegicus Aging. *Cell* 180 984–1001.e22. 10.1016/j.cell.2020.02.008 32109414

[B37] Macut D. Bjekic-Macut J. Rahelic D. Doknic M. (2017). Insulin and the polycystic ovary syndrome. *Diabetes Res. Clin. Pract.* 130 163–170. 10.1016/j.diabres.2017.06.011 28646699

[B38] Mao Z. Li T. Zhao H. Qin Y. Wang X. Kang Y. (2021). Identification of epigenetic interactions between microRNA and DNA methylation associated with polycystic ovarian syndrome. *J. Hum. Genet.* 66 123–137. 10.1038/s10038-020-0819-6 32759991 PMC7782128

[B39] Merkin S. S. Phy J. L. Sites C. K. Yang D. (2016). Environmental determinants of polycystic ovary syndrome. *Fertil. Steril.* 106 16–24. 10.1016/j.fertnstert.2016.05.011 27240194

[B40] Moran L. J. Misso M. L. Wild R. A. Norman R. J. (2010). Impaired glucose tolerance, type 2 diabetes and metabolic syndrome in polycystic ovary syndrome: a systematic review and meta-analysis. *Hum. Reprod. Update* 16 347–363. 10.1093/humupd/dmq001 20159883

[B41] Mu L. Sun X. Tu M. Zhang D. (2021). Non-coding RNAs in polycystic ovary syndrome: a systematic review and meta-analysis. *Reprod. Biol. Endocrinol.* 19:10. 10.1186/s12958-020-00687-9 33446212 PMC7807442

[B42] Muneeb A. F. Rima D. Reetika S. Daman S. Tanuj D. (2014). CYP1B1: a Unique Gene with Unique Characteristics. *Curr. Drug Metab.* 15 893–914. 10.2174/1389200216666150206130058 25658124

[B43] Mutharasan P. Galdones E. Penalver Bernabe B. Garcia O. A. Jafari N. Shea L. D. (2013). Evidence for chromosome 2p16.3 polycystic ovary syndrome susceptibility locus in affected women of European ancestry. *J. Clin. Endocrinol. Metab.* 98 E185–90. 10.1210/jc.2012-2471 23118426 PMC3537106

[B44] Ni W. Zhang S. Jiang B. Ni R. Xiao M. Lu C. (2019). Identification of cancer-related gene network in hepatocellular carcinoma by combined bioinformatic approach and experimental validation. *Pathol. Res. Pract.* 215:152428. 10.1016/j.prp.2019.04.020 31064721

[B45] Pan M. L. Chen L. R. Tsao H. M. Chen K. H. (2015). Relationship between Polycystic Ovarian Syndrome and Subsequent Gestational Diabetes Mellitus: a Nationwide Population-Based Study. *PLoS One* 10:e0140544. 10.1371/journal.pone.0140544 26488176 PMC4619482

[B46] Paravati R. De Mello N. Onyido E. K. Francis L. W. Brüsehafer K. Younas K. (2020). Differential regulation of osteopontin and CD44 correlates with infertility status in PCOS patients. *J. Mol. Med.* 98 1713–1725. 10.1007/s00109-020-01985-w 33047155 PMC7679339

[B47] Park J. S. Lee H. Choi B. W. Ro S. Lee D. Na J. E. (2018). - An MG53-IRS1-interaction disruptor ameliorates insulin resistance. *Exp. Mol. Med.* 50 1–12. 10.1038/s12276-018-0099-9 29884820 PMC5994830

[B48] Patil K. Hinduja I. Mukherjee S. (2020). Alteration in angiogenic potential of granulosa-lutein cells and follicular fluid contributes to luteal defects in polycystic ovary syndrome. *Hum. Reprod*. 36 1052–1064. 10.1093/humrep/deaa351 33377483

[B49] Ping H. Siqi B. Dandan F. Congcong Y. Jianzhong S. Jia Q. (2020). Machine learning-based integrative analysis of methylome and transcriptome identifies novel prognostic DNA methylation signature in uveal melanoma. *Brief. Bioinform.* 1–12. 10.1093/bib/bbaa371 [Epub Online ahead of print]. 33367533

[B50] Qi X. Yun C. Sun L. Xia J. Wu Q. Wang Y. (2019). Gut microbiota–bile acid–interleukin-22 axis orchestrates polycystic ovary syndrome. *Nat. Med.* 25 1225–1233. 10.1038/s41591-019-0509-0 31332392 PMC7376369

[B51] Reece A. S. Hulse G. K. (2020). Canadian Cannabis Consumption and Patterns of Congenital Anomalies: an Ecological Geospatial Analysis. *J. Addict. Med.* 14 e195–e210. 10.1097/ADM.0000000000000638 32187114 PMC7547880

[B52] Roth L. W. McCallie B. Alvero R. Schoolcraft W. B. Minjarez D. Katz-Jaffe M. G. (2014). Altered microRNA and gene expression in the follicular fluid of women with polycystic ovary syndrome. *J. Assist. Reprod. Genet.* 31 355–362. 10.1007/s10815-013-0161-4 24390626 PMC3947080

[B53] Salehi Jahromi M. Hill J. W. Ramezani Tehrani F. Zadeh-Vakili A. (2018). Hypomethylation of specific CpG sites in the promoter region of steroidogeneic genes (GATA6 and StAR) in prenatally androgenized rats. *Life Sci.* 207 105–109. 10.1016/j.lfs.2018.05.052 29859221

[B54] Shannon P. Markiel A. Ozier O. Baliga N. S. Wang J. T. Ramage D. (2003). Cytoscape: a software environment for integrated models of biomolecular interaction networks. *Genome Res.* 13 2498–2504. 10.1101/gr.1239303 14597658 PMC403769

[B55] Sirotkin A. V. Laukova M. Ovcharenko D. Brenaut P. Mlyncek M. (2010). Identification of microRNAs controlling human ovarian cell proliferation and apoptosis. *J. Cell Physiol.* 223 49–56. 10.1002/jcp.21999 20039279

[B56] Song J. Diao F. Ma X. Xu S. Cui Y. Jiang S. (2019). Androgen upregulates NR4A1 via the TFAP2A and ETS signaling networks. *Int. J. Biochem. Cell Biol.* 113 1–7. 10.1016/j.biocel.2019.05.015 31146003

[B57] Sun J. Zhang Z. Bao S. Yan C. Hou P. Wu N. (2020). Identification of tumor immune infiltration-associated lncRNAs for improving prognosis and immunotherapy response of patients with non-small cell lung cancer. *J. Immunother. Cancer* 8:e000110. 10.1136/jitc-2019-000110 32041817 PMC7057423

[B58] Vlachos I. S. Paraskevopoulou M. D. Karagkouni D. Georgakilas G. Vergoulis T. Kanellos I. (2015). DIANA-TarBase v7.0: indexing more than half a million experimentally supported miRNA:mRNA interactions. *Nucleic Acids Res.* 43 D153–D159. 10.1093/nar/gku1215 25416803 PMC4383989

[B59] Wang T. Liu Y. Lv M. Xing Q. Zhang Z. He X. (2019). miR-323-3p regulates the steroidogenesis and cell apoptosis in polycystic ovary syndrome (PCOS) by targeting IGF-1. *Gene* 683 87–100. 10.1016/j.gene.2018.10.006 30300681

[B60] Yu G. Wang L.-G. He Q.-Y. (2015). ChIPseeker: an R/Bioconductor package for ChIP peak annotation, comparison and visualization. *Bioinformatics* 31 2382–2383. 10.1093/bioinformatics/btv145 25765347

[B61] Yu Y. Y. Sun C. X. Liu Y. K. Li Y. Wang L. Zhang W. (2015). Genome-wide screen of ovary-specific DNA methylation in polycystic ovary syndrome. *Fertil. Steril.* 104 145–153.e6. 10.1016/j.fertnstert.2015.04.005 25956362

[B62] Zhang Y. Meng N. Bao H. Jiang Y. Yang N. Wu K. (2019). Circadian gene PER1 senses progesterone signal during human endometrial decidualization. *J. Endocrinol.* 243 229–242. 10.1530/JOE-19-0284 31518992

[B63] Zhao H. Zhao Y. Ren Y. Li M. Li T. Li R. (2017). Epigenetic regulation of an adverse metabolic phenotype in polycystic ovary syndrome: the impact of the leukocyte methylation of PPARGC1A promoter. *Fertil. Steril.* 107 467–474.e5. 10.1016/j.fertnstert.2016.10.039 27889100

[B64] Zhou M. Zhang Z. Bao S. Hou P. Sun J. (2020). Computational recognition of lncRNA signature of tumor-infiltrating B lymphocytes with potential implications in prognosis and immunotherapy of bladder cancer. *Brief. Bioinform.* 1–13. 10.1093/bib/bbaa047 [Epub online ahead of print]. 32382761

