# Supplementary material for: Characterization of DNA Methylation and Screening of Epigenetic Markers in Polycystic Ovary Syndrome
Source: Front Cell Dev Biol. 2021 May 25;9:664843. doi: 10.3389/fcell.2021.664843 (PMC8186667; doi:10.3389/fcell.2021.664843)
Supplement: Supplementary file 1 [file Data_Sheet_1.PDF]

Supplementary Figure 1

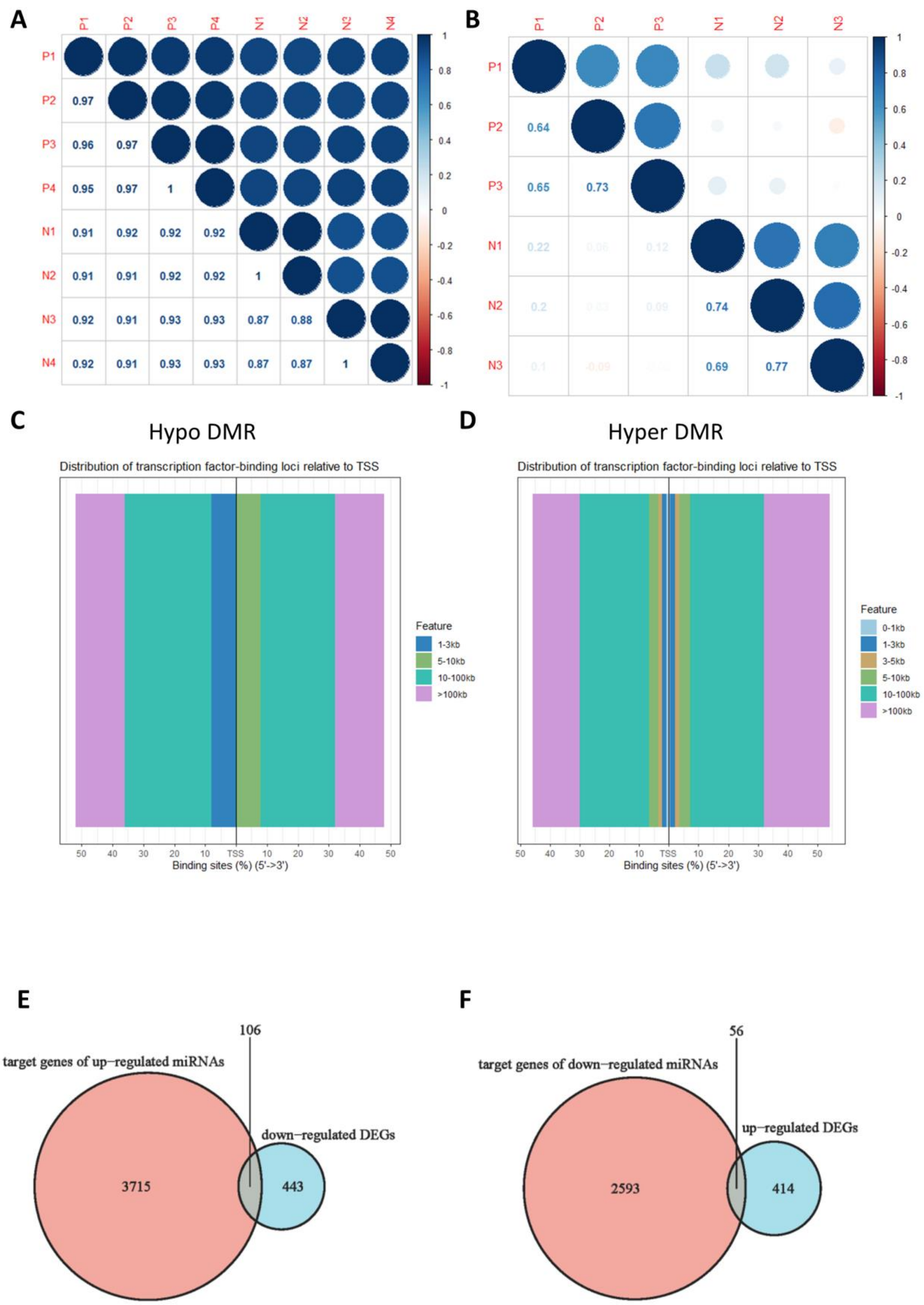

Supplementary Figure 1

(A, B) Correlation analysis of transcriptome and DNA methylayion samples, respectively. (C, D) The distance from transcription initiation site of hypo DMRs and hyper DMRs, respectively. (E, F)

Venn diagram showing the overlap of target genes of the differentially expressed miRNAs and differentially expressed genes, respectively.
